# Symptoms and signs associated with benign and malignant proximal fibular tumors: a clinicopathological analysis of 52 cases

**DOI:** 10.1186/s12957-017-1162-z

**Published:** 2017-05-02

**Authors:** Tao Sun, Lingxiang Wang, Changzhi Guo, Guochuan Zhang, Wenhai Hu

**Affiliations:** 1 Department of Orthopaedic Oncology, Hebei Medical University Third Affiliated Hospital, 139 Ziqiang Road, Shijiazhuang, 050051 Hebei Province China; 2 Department of Gynecology, Hebei Medical University Fourth Affiliated Hospital, Shijiazhuang, 050011 Hebei Province China

**Keywords:** Proximal fibular, Benign, Malignant, Bone tumor, Symptom and sign

## Abstract

**Background:**

Malignant tumors in the proximal fibula are rare but life-threatening; however, biopsy is not routine due to the high risk of peroneal nerve injury. Our aim was to determine preoperative clinical indicators of malignancy.

**Methods:**

Between 2004 and 2016, 52 consecutive patients with proximal fibular tumors were retrospectively reviewed. Details of the clinicopathological characteristics including age, gender, location of tumors, the presenting symptoms, the duration of symptoms, and pathological diagnosis were collected. Descriptive statistics were calculated, and univariate and multivariate regression were performed.

**Results:**

Of these 52 patients, 84.6% had benign tumors and 15.4% malignant tumors. The most common benign tumors were osteochondromas (46.2%), followed by enchondromas (13.5%) and giant cell tumors (13.5%). The most common malignancy was osteosarcomas (11.5%). The most common presenting symptoms were a palpable mass (52.0%) and pain (46.2%). Pain was the most sensitive (100%) and fourth specific (64%); both high skin temperature and peroneal nerve compression had the highest specificity (98%) and third sensitivity (64%); change in symptoms had the second highest specificity (89%) while 50% sensitivity. Using multivariate regression, palpable pain, high skin temperature, and peroneal nerve compression symptoms were predictors of malignancy.

**Conclusions:**

Most tumors in the proximal fibula are benign, and the malignancy is rare. Palpable pain, peroneal nerve compression symptoms, and high skin temperature were specific in predicting malignancy.

## Background

The primary fibular tumor is rare with only 2.5% of all primary bone tumors occurring in this anatomical location [1]. The proximal fibula is the most common area of the fibula to be affected by tumors, and osteosarcoma, giant cell tumors, chondrosarcoma, and aneurysmal bone cysts are the most common type of tumor to develop at this location [2]. Although, most proximal fibular tumors are benign; however, malignant tumors account for a significant amount of morbidity and mortality. The diagnosis of proximal fibular malignant bone tumors is hampered by delays in presentation.

Most patients with symptomatic benign tumors or malignant tumors in the proximal fibula require surgical management. Intralesional or marginal excision was often performed in benign tumors, while en bloc resection is recommended to be performed in aggressive benign tumors and malignant tumors [3–5]. The preoperative chemotherapy is based on biopsy results and plays an important role in prognosis of malignant bone tumors, especially osteosarcoma [6]. Given the sensitive anatomy in this location, biopsy is not considered unless malignancy is highly suspected. It is necessary, therefore, to obtain more information of symptoms and signs in predicting benign or malignant proximal fibular tumors.

The differences in clinical presentation and medical images between benign and malignant proximal fibular tumors are not well recognized given the paucity of literature. It is for this reason that we retrospectively reviewed proximal fibular cases with pathological diagnosis to determine preoperative indicators of benign or malignant tumors.

## Methods

We performed a retrospective review of our institution’s pathologic and surgical databases from 2004 to 2016 to identify all patients with proximal fibular tumors that had been confirmed histologically and treated surgically. This study has been approved by the Institutional Review Board. Written informed consent were obtained from the participants. While the patients were not specifically recalled for the study, the medical records, radiographs, and histological specimens of each patient were analyzed.

We identified 52 patients with proximal fibular tumors who were diagnosed and treated in our institute during this time. Those who were initially treated elsewhere and referred due to a recurrence, as well as none operative cases, were excluded. Details of the clinicopathological characteristics including age, gender, location of tumors, the presenting symptoms, the duration of symptoms, and pathological diagnosis were reviewed and compared using ANOVA for continuous variables and chi-square test or Fisher’s exact tests for categorical data.

First, malignant tumors were compared with benign tumors using the descriptive statistics of sensitivity and specificity; positive predictive value (PPV) and negative predictive value (NPV) were calculated for each variable. Univariate and multivariate logistic regressions were then performed to identify predictors associated with malignancy. Statistical analysis was performed by using SPSS 19.0 (SPSS, Inc., Chicago, IL, USA).

## Results

### Patient characteristics

All diagnoses were histologically confirmed (Table 1). Tumors were classified according to the Musculoskeletal Tumor Society [7, 8]. There were 26 males and 26 females with a mean age of 26.5 years (range, 4–72 years). The proximal epiphysis was involved in 12 patients (23.1%). The metaphyseal region of the proximal fibula was implicated in 28 patients (53.8%). Both the epiphysis and metaphyseal regions of the proximal fibula were involved in 12 patients (23.1%). The tumors were located on the right side in 18 patients (34.6%) and the left side in 34 patients (65.4%).

**Table 1:** Histologic diagnoses and clinical characteristics of tumors of the proximal part of the fibula

Diagnosis		Number of case	Symptoms and signs							Onset of tumors (month)
			Pain	Palpable mass	Imaging examination	Palpable pain	High temperature	Peroneal nerve compression	Changes in symptoms	
Benign	Osteochondroma	24 (46.2%)	3	21	1	3	–	–	1	14.4
	Enchondroma	7 (13.5%)	2	1	5	1	–	–	1	12.4
	Giant cell tumor	7 (13.5%)	5	3	1	5	1	–	1	3.0
	Chondroblastoma	2 (3.8%)	2	–	–	–	–	1	1	4.0
	Osteoblastoma	2 (3.8%)	2	–	–	–	–	–	–	6.5
	Osteoid osteoma	1 (1.9%)	1	–	–	–	–	–	–	2.0
	Aneurysmal bone cyst	1 (1.9%)	1	–	–	–	–	–	1	36.0
Malignant	Osteosarcoma	6 (11.5%)	6	2	–	4	5	5	3	3.7
	Chondrosarcoma	1 (1.9%)	1	–	–	1	–	–	–	1.0
	Metastatic bone disease	1 (1.9%)	1	–	–	1	–	–	1	0.5
Total no. of signs and symptoms		52 (100%)	24 (46.2%)	27 (52.0%)	7 (13.5%)	15 (28.8%)	6 (11.5%)	6 (11.5%)	9 (17.3%)	10.31

**Fig. 1:**
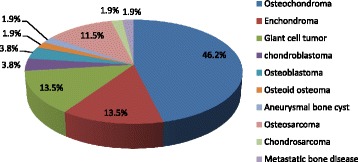
Histologic types of proximal fibular tumor

All 52 proximal fibular tumors were histologically confirmed by the pathologist (Fig. 1), while slides were not re-reviewed for the current study. Forty-four patients had benign tumors (84.6%) and 8 had malignant tumors (15.4%). Osteochondromas were the most common benign proximal fibular tumors (24 cases, 46.2%), followed by enchondromas in 7 cases (13.5%) and giant cell tumors in 7 cases (13.5%) including 3 cases associated with aneurysmal bone cyst. The most common malignant tumor was osteosarcoma in 6 patients (11.5%).

Clinical characteristics of the patients with the proximal fibular tumors are shown in Table 1. A palpable mass was the most common presenting symptom (27 cases, 52.0%) followed by pain in 24 patients (46.2%) and by imaging examination in 7 patients (13.5%). Five patients (9.6%) presented with signs and/or symptoms of peroneal nerve compression. Nine cases (17.3%) presented due to change of symptoms. Except for signs of palpable mass and peroneal nerve compression, the common signs included palpable pain (15 cases, 28.8%) and increased skin temperature (6 cases, 11.5%). Patients came to clinic 10.31 months in average (range, 2 h to 9 years) after onset of tumors. The presenting symptoms were considered to be those specifically told to the surgeon by the patient, as documented in the medical record.

All cases included in this study had surgical treatment (Table 2). Intralesional excision of tumor was performed in 4 patients (7.7%), marginal excision in 22 patients (42.3%), and en bloc resection in 26 patients (50.0%), and there is no amputation case in this study. Four cases of core biopsy and 2 cases of incision biopsy had been performed before the definite surgeries. The most common indications for intralesional treatment were enchondroma, osteoblastoma, and osteoid osteoma. Marginal resections were performed for enchondroma. En bloc resection was most commonly performed for aggressive benign tumors, such as epiphyseally located giant cell tumors, aneurysmal bone cysts, enchondromas, and osteochondromas, and all malignant tumors (Table 2). Of the 26 en bloc proximal fibula resections, type I proximal fibula resection was done in 22 cases and type II in 4 cases per Malawer’s description [4].

**Table 2:** Surgical treatment of 52 bone tumors of the proximal part of the fibula

Diagnosis		Surgical intervention (no.)				Total tumors by diagnosis (*n* = 52)
		Intralesional excision (*n*=)	Marginal excision (*n*=)	Type-1 en bloc resection (*n*=)	Type-2 en bloc resection (*n*=)	
Benign	Osteochondroma	–	22	2	–	24 (46.2%)
	Enchondroma	2	–	5	–	7 (13.5%)
	Giant cell tumor	–	–	7	–	7 (13.5%)
	Chondroblastoma	–	–	2	–	2 (3.8%)
	Osteoblastoma	1	–	1	–	2 (3.8%)
	Osteoid osteoma	1	–	–	–	1 (1.9%)
	Aneurysmal bone cyst	–	–	1	–	1 (1.9%)
Malignant	Osteosarcoma	–	–	2	4	6 (11.5%)
	Chondrosarcoma	–	–	1	–	1 (1.9%)
	Metastatic bone disease	–	–	1	–	1 (1.9%)
Total tumors by surgical intervention (no.)		4 (7.7%)	22 (42.3%)	22 (42.3%)	4 (7.7%)	52 (100%)

### Benign vs. malignant proximal fibular tumors

Descriptive statistics were calculated for several variables and are shown in Table 3. The differences in pain, palpable pain, high local skin temperature, peroneal nerve compression, and changes in symptoms between benign and malignant proximal fibular tumors were statistically significant (*P* < 0.05). Pain was the most sensitive (100%) and fourth specific (64%) for the presence of malignancy. A patient presenting with pain had an almost threefold greater chance of malignant than benign lesions. Both high skin temperature and peroneal nerve compression had the highest specificity (98%) and third sensitivity (64%). Their positive likelihood ratio is 27.5, which suggests that the above symptoms and signs, when present, increase the likelihood of malignancy 27.5 times relative to begin lesions. Change in symptoms had the second highest specificity (89%) while 50% sensitivity. When present with changed symptoms, there was a 4.4-fold greater chance that lesion was malignant as compared with benign lesions. Other clinical findings did not result in meaningful improvements in sensitivity or specificity for malignancy (Table 3).

**Table 3:** Descriptive statistics for predictors of malignancy

Variable	Benign (*n* = 44)	Malignant (*n* = 8)	Statistic value	*P* value	Sens.	Spec.	PPV	NPV	LR+	LR−
Age mean (SD)	24.7 (16.4)	36.6 (23.4)	*F* = 3.118	0.084	N/A	N/A	N/A	N/A	N/A	N/A
Male	23	3	*χ* ^2^ = 0.148	0.701	38%	48%	12%	48%	0.72	1.31
Left	28	6	*χ* ^2^ = 0.047	0.828	75%	36%	18%	36%	1.18	0.69
Pain	16	8	*χ* ^2^ = 8.618	0.003	100%	64%	33%	64%	2.75	N/A
Palpable mass	25	3	*χ* ^2^ = 0.388	0.533	38%	43%	11%	43%	0.66	1.45
Imaging examination	7	0	*χ* ^2^ = 0.422	0.516	0%	84%	0%	84%	0	1.19
Palpable pain	9	6	*χ* ^2^ = 7.335	0.007	5%	60%	25%	60%	0.14	1.58
High temperature	1	5	*χ* ^2^ = 24.056	<0.001	63%	98%	83%	98%	27.50	0.38
Peroneal nerve compression	1	5	*χ* ^2^ = 24.056	<0.001	63%	98%	83%	98%	27.50	0.38
Changes in symptom	5	4	*χ* ^2^ = 7.060	0.008	50%	89%	44%	89%	4.40	0.56
Duration month mean (SD)	11.7 (20.3)	2.9 (2.5)	*F* = 1.448	0.235	N/A	N/A	N/A	N/A	N/A	N/A

*LR+* positive likelihood ratio, *LR–* negative likelihood ratio, *NPV* negative predictive value, *PPV* positive predictive value, *N/A* not applicable

Next, we utilized univariate and multivariate liner regression to identify predictors of malignancy (Table 4). Pain, palpable pain, high temperature, peroneal nerve compression, and change in symptom were significant in the univariate analysis. However, when entered into the multivariate model, palpable pain, high temperature, and peroneal nerve compression were predictive for malignancy.

**Table 4:** Liner regression analysis of preoperative clinical predictors of malignancy

Variable	Univariate *P* value	Multivariate *P* value
Pain	0.001	0.971
Palpable pain	0.001	0.025
High temperature	<0.001	0.007
Peroneal nerve compression	<0.001	0.003
Change in symptom	0.007	0.524

## Discussion

The proximal fibula can develop all types of benign or malignant bone tumors seen in the rest of the human skeleton. Although the most common benign and malignant tumors in the proximal fibula are osteochondroma and osteosarcoma, respectively, according to studies by both the Mayo Clinic and our institute [9, 10], the ratio of benign to malignancy is still not well defined. A study reported that approximately one third of all tumors in this anatomic location are benign [1]. However, Abdel et al. reported 121 benign to 112 malignant proximal fibular tumors in the Mayo series [9, 10]. In this study, the benign to malignant ratio was 5.5:1 and this may still be underestimated, because the above studies included only patients that underwent surgery, but excluded those with benign tumors who abandoned surgery.

Although most proximal fibular tumors are benign, malignant tumors are rare but life-threatening. It is well recognized that finding out the presenting symptoms and signs which can predict malignancy plays an important role in the treatment. Unfortunately, the symptoms and signs were quite different among patients, including pain, palpable mass, pathologic fracture, restricted knee motion, local swelling, and symptoms of peroneal nerve compression, as well as palpable mass or pain and higher skin temperature and changes in symptoms or signs [9, 11]. Although pain was the most common symptom [9, 11], the sensitivity and specificity of the above symptoms and signs were not clarified. Base on our study, pain was the most sensitive symptom but not so specific. In addition, palpable pain, peroneal nerve compression symptoms, and high skin temperature were specific, while relatively sensitive. A study, including 13 cases of osteosarcoma in the proximal fibula, reported the duration of symptoms before consulting a doctor varied, ranging from 1 to 6 months with a median of 2 months [11]. In the present study, the average duration is 10.3 months with a range from 2 h to 9 years. Although the average duration of benign tumors is 11.7 months, whereas that of malignant was 2.9 months, the difference between benign and malignant tumors was not significant. Therefore, similar to gender and limb side, the duration cannot be used to predict malignancy.

In the present study, 6 patients (11.5%) underwent core biopsy or incisional biopsy. A study in the Mayo clinic reported 8% of patients with proximal fibular malignant tumors had an incisional biopsy followed by radiation therapy and/or chemotherapy [10]. Another study in Japan reported 46% (6/13) osteosarcomas in the proximal fibula received preoperative chemotherapy after biopsy [11]. Not all malignant bone tumors in the proximal fibula can undergo biopsy and receive preoperative chemotherapy, which may adversely affect limb salvage surgery and prognosis. Biopsy is not routinely performed in the diagnosis of proximal fibular tumors because the superficial and deep peroneal nerves are the most important structures in relation to bone tumors of the proximal fibula and may be damaged during biopsy. An anatomical study suggested the biopsy should be performed by an anterolateral approach through the safe area in the compartment of the peroneus longus muscle bounded by the head of the fibula and the deep peroneal nerve [12]. In our experience, core biopsy can performed on the tumor confined to the fibular head under X-ray guidance, and incision biopsy is recommended when the tumor involves both the epiphyseal and metaphyseal regions. It is safe and necessary to perform biopsy on proximal fibular tumors when suspecting malignancy.

When surgical management is concerned, most of benign proximal fibular tumors were managed by intralesional or marginal excision, while malignant tumors required aggressive surgical management with radical or wide resection [10, 13]. For osteosarcoma and Ewing sarcoma, pre- and postoperative radiation therapy and/or chemotherapy were equally important [10, 11]. Although an above-the-knee amputation has not been performed in our case series, amputation is a kind of radical resection, which was led by diagnoses of osteosarcoma, Ewing sarcoma, fibrosarcoma, hemangiosarcoma, chondrosarcoma, and metastatic disease, or as a result of postoperative complications, such as recurrent infection [10]. The amputation rate decreased recently, while the type-II en bloc resection increased in surgical treatment of malignancy in the proximal fibula [10]. This trend may result from advances in surgical techniques and early diagnosis of malignancy by medical imaging test. The main positive complications included instable keen, permanent peroneal nerve palsies, local recurrences, and thrombosis of the posterior tibial artery, skin necrosis, and wound-healing failure [10, 11].

The present study is limited by only including patients who received surgery and had histologic diagnosis. The benign to malignancy ratio may be underestimated due to not including those who abandoned surgery. This study determined the association of symptoms and signs of malignancy; therefore, further research concerning the relationship between different surgeries and complications should be carried out.

## Conclusions

Although the incidence of malignant tumors is much lower than that of benign tumors in the proximal fibula, a biopsy should be considered when patients presented with palpable pain, peroneal nerve compression symptoms, and high skin temperature, which were specific in predicting malignancy.
